# Simplifying the diagnosis of optic tract lesions

**DOI:** 10.3389/fmed.2022.1029829

**Published:** 2022-10-25

**Authors:** Noa Cohen-Sinai, Inbal Man-Peles, Alon Zahavi, Judith Luckman, Nitza Goldenberg-Cohen

**Affiliations:** ^1^Department of Ophthalmology, Bnai Zion Medical Center, Haifa, Israel; ^2^Department of Ophthalmology, Rabin Medical Center, Petach Tikva, Israel; ^3^Sackler Faculty of Medicine, Tel Aviv University, Tel Aviv, Israel; ^4^Department of Radiology, Rabin Medical Center, Petach Tikva, Israel; ^5^Bruce and Ruth Faculty of Medicine, Technion, Israel Institute of Technology, Haifa, Israel; ^6^The Krieger Eye Research Laboratory, Faculty of Medicine, Technion, Israel Institute of Technology, Haifa, Israel

**Keywords:** optic tract lesions, optical coherence tomography, visual field, magnetic resonance imaging, visual pathway

## Abstract

Optic tract lesions (OTL) are often difficult to diagnose. We suggest an algorithm to simplify the often-challenging diagnosis of OTL. Clinical and imaging data were retrospectively collected from the electronic files of 6 patients diagnosed with OTL at a tertiary medical center in 2016–2020. The series included 4 children and 2 adults with an OTL caused by a glioma (*n* = 5) or motor vehicle accident (*n* = 1). Magnetic resonance imaging (MRI) revealed a suprasellar glioma involving the chiasm and tract alone (n = 1) and the ipsilateral optic nerve (n = 2) and only optic tract (3). Perimetry showed incongruent homonymous hemianopia in 3 patients. In two patients, perimetry could only be performed in one eye, and demonstrated hemianopia. In one patient perimetry was unreliable. Fundus examination revealed bow-tie atrophy in all patients. On optical coherence tomography (OCT) of the peripapillary retinal nerve fiber layer (RNFL) horizontal thinning was observed in the contralateral eye (*n* = 6). By presenting the information in a predefined order—visual field damage, OCT RNFL thickness, and MRI—the diagnosis could be easily reached even in children, and when other structures like the chiasm were involved. Fundus photographs easily detect bow tie atrophy in children. Systematic presentation of the data in a predefined order can ease the diagnostic process of OTLs.

## Introduction

Optic tract lesions (OTL) are often difficult to diagnose. They are usually due to a mass lesion such as a tumor or aneurysm (1), and less frequently to trauma (2) or inflammatory or demyelinating disease (3, 4). Clinical manifestations include optic tract syndrome with incongruous homonymous hemianopia, visual field defects (5), relative afferent pupillary defect (RAPD) of the contralateral eye, and optic atrophy (1, 5). Some patients present with complete homonymous hemianopia or monocular visual loss with hemianopia in the contralateral eye. When optic atrophy develops, a unique pathognomonic bow-tie appearance of relative horizontal pallor of the contralateral optic disc can be seen (1). Although all data exist, it is hard to complete the diagnostic puzzle. Anatomical evaluation of the optic nerve head including ancillary tests such as color photography of the optic nerve head, ocular ultrasonography (US), and optical coherence tomography (OCT) scans of the peripapillary retinal nerve fiber layer (RNFL) may help clinicians recognizing specific and sometimes subtle RNFL defect patterns such as the bow-tie sign. However, the use of US or OCT in the diagnosis of OTL in patients with hemianopia has hardly been investigated (6–11). In suspected cases, magnetic resonance imaging (MRI) scans of the head directed at the optic tract are used to locate the lesion (5, 12). The correlation of the clinical, visual field, OCT of RNFL and retinal ganglion cells (RGCs), and MRI findings confirm the diagnosis.

The aim of the present study was to present an algorithm to simplify the diagnosis of OTL based on integration of the findings of the different modalities available.

## Materials and methods

A retrospective chart review study design was used. The medical database of the ophthalmology department was reviewed for all patients diagnosed with optic neuropathy, with suspected OTL from 2016 to 2020. Data on demographics, clinicopathological findings, imaging scans, surgical procedures, and outcomes were collected from the electronic medical files. Visual function was assessed clinically, including visual acuity, color vision (using Ishihara color plates), RAPD, and optic disc appearance. Visual field testing was performed with a Humphrey Field Analyzer (Carl Zeiss Meditec) combined with the Swedish Interactive Threshold Algorithm standard 24-2 program, as part of the functional assessment of the optic nerve. Results were interpreted by a senior neuro-ophthalmologist. OCT scans were performed with a spectral domain OCT system (Cirrus 4000, Carl Zeiss Meditec, Dublin, CA, USA or Spectralis, Heidelberg Engineering, Franklin, MA, USA), and the results were reviewed for RNFL defects and retinal ganglion cell (RGC) loss. Fundus photographs were taken with a DRSplus confocal fundus imaging system (CenterVue Inc., Fremont, CA, USA). MRI scans were reviewed for OTL.

The study was conducted in accordance with the tenants of the declaration of Helsinki with approval by the local Institutional Review Board.

## Results

Six patients met the study criteria, 2 female and 4 male, of mean age 20.67 ± 18.4 years (2 adults and 4 children). Five had glioma of the optic tract, 3 with chiasmal involvement, and one had post-traumatic OTL; signs in all 6 were similar. Only when combining the data in a predefined order, including a high quality fundus photography of the optic disc, the diagnosis of the OTL becomes clear even in the early phases of evaluation.

### Case 1

A 26-year-old female sustained severe blunt head trauma with loss of consciousness in a motor vehicle accident. Computerized tomography of the head showed multiple hematomas, bilaterally in the frontal lobe and in the right parietal lobe, right temporal lobe, pontine, and cerebellum. She was sedated and intubated, and frontal burr hole craniotomy was performed.

Two months later, during recovery, the patient complained of a left visual field disturbance. Examination revealed best corrected visual acuity (BCVA) 6/6 in each eye, RAPD + 2 in the left eye, intact Ishihara color vision test, left complete homonymous visual field defect on confrontation, and normal ocular movements, anterior segments, and intraocular pressure. Disc appearance was normal on fundoscopy in both eyes. Humphrey automated perimetry confirmed complete left homonymous hemianopia.

**FIGURE 1:**
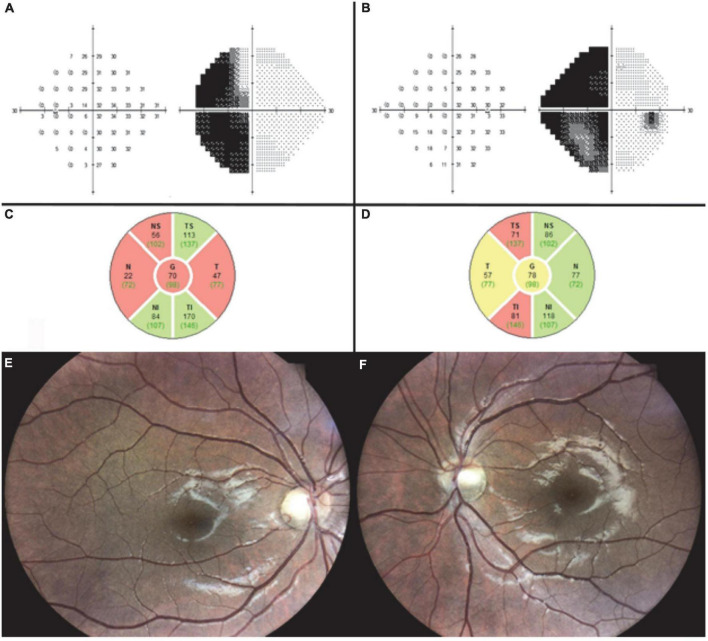
Case 1. Ten months after traumatic injury, automated perimetry shows unchanged left homonymous hemianopia in the left **(A)** and right **(B)** eyes. OCT shows RNFL loss in the left temporal and nasal quadrants **(C)** and the right superior and inferior quadrants **(D)**. Fundus imaging 3 years after injury shows right optic disc temporal pallor **(E)** and left optic disc bow-tie atrophy **(F)**.

MRI performed at 9 months after injury revealed signs of diffuse axonal injury and right temporal gliosis adjacent to the right optic tract. At 10 months, optic disc examination revealed bow-tie atrophy of the left optic disc and temporal pallor of the right optic disc, with no change in automated perimetry findings. OCT demonstrated RNFL loss in the right superior and inferior quadrants and in the left temporal and nasal quadrants (Figures 1A–D). The combined clinical and imaging data confirmed the diagnosis of right optic tract syndrome.

Fundus imaging 3 years after injury demonstrated the typical pattern of left optic disc bow-tie atrophy and right optic disc temporal pallor (Figures 1E,F).

### Case 2

**FIGURE 2:**
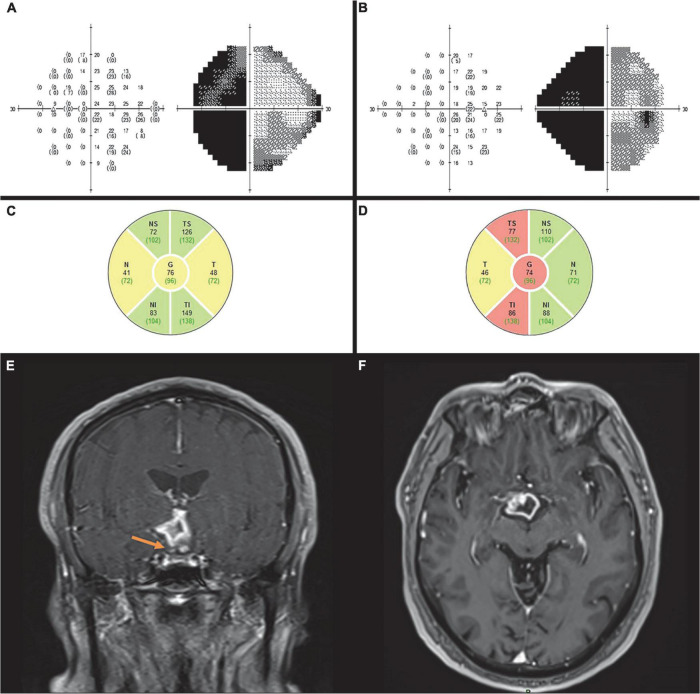
Case 2. Automated perimetry shows left homonymous hemianopia in the left **(A)** and right **(B)** eye. OCT demonstrates RNFL thinning in the left nasal and temporal **(C)** and right superior and inferior quadrants **(D)**. MRI T1 coronal **(E)** and axial imaging. **(F)** After contrast show a ring enhancing lesion involving the hypothalamus and right optic tract (arrow) causing posterior compression of the chiasm consistent with low -grade glioma.

A 59-year-old male presented with complaints of longstanding headaches and visual impairment. On examination, BCVA was 6/7.5 in both eyes and Ishihara color vision test was normal. Anterior segments, intraocular pressure, and eye movements were normal bilaterally. Positive RAPD was noted in the left eye, and fundoscopy revealed relative pallor of the left disc. Humphrey automated perimetry test showed left homonymous hemianopia bilaterally (Figures 2A,B). On OCT, there was a significant RNFL thinning in the superior and inferior quadrants of the right eye and in the nasal and temporal quadrants of the left eye (Figures 2C,D). The clinical findings suggested OTL. The diagnosis was confirmed by MRI which revealed a right suprasellar space-occupying lesion compressing the right optic tract (Figures 2E,F). The lesion was surgically removed in a transcallosal interhemispheric approach and was characterized pathologically as pilocytic astrocytoma.

### Case 3

**FIGURE 3:**
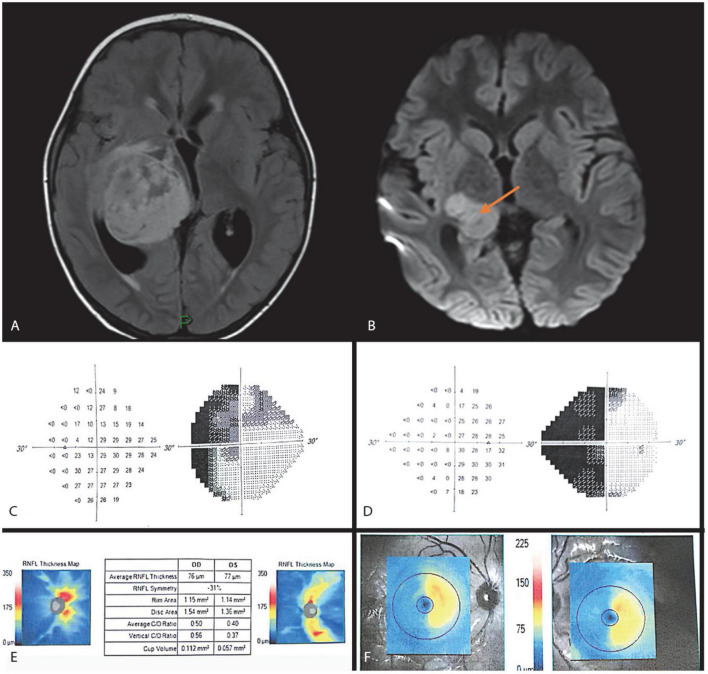
Case 3. MRI FLAIR and DWI images **(A,B)** show a right thalamic lesion extending to the right optic radiation causing significant mass effect and hydrocephalus. Automated perimetry at 12 months after surgery showing left homonymous hemianopia **(C,D)**. OCT 6 months after surgery showing RNFL thinning in the right superior and inferior and left nasal and temporal quadrants **(E),** and related retinal ganglion cell counts **(F)**.

A 6-year-old boy was referred to our clinic because of positive left RAPD found incidentally on routine examination. Anisometropic amblyopia of the left eye did not explain the RAPD. MRI demonstrated a large thalamic glioma in the right hemisphere (Figures 3A,B). The glioma was surgically removed, and adjuvant vincristine and carboplatin chemotherapy was administered. Visual acuity prior to surgery was 6/9 in the right eye and 6/18 in the left eye, partially attributed to anisometropia, hypermetropia, and amblyopia. He was treated with glasses, and his corrected VA improved to 6/6 in each eye, by the end of follow up.

On follow-up examination 3 months postoperatively, left RAPD was still positive and BCVA was 6/9 in the right eye and 6/21 in the left eye; corresponding cycloplegic refraction was + 3.5D and + 5.0D. Anterior segments, eye movements, and intraocular pressure were normal bilaterally. Results of partial Ishihara color vision test were 4/10 in each eye.

OCT performed 6 months after surgery showed RNFL loss in both the nasal and temporal quadrants in the left eye and the superior and inferior quadrants in the right eye (Figure 3E). Humphrey automated perimetry at 12 months demonstrated left homonymous hemianopia (Figures 3C,D). Temporal left disc pallor was prominent on fundoscopy. Additionally, a GCL OCT scan was performed demonstrating thinning of the GCL layer nasally in the right eye and temporally in the left, confirming the VF findings (Figure 3F).

### Case 4

A 14-year-old boy with genetically confirmed neurofibromatosis type 1 presented with blurred vision in the right eye of 2 weeks’ duration with no other neurological or systemic complaints. Past medical history was remarkable for a longstanding chiasmatic glioma causing severe left optic atrophy and obstructive hydrocephalus which was treated with a ventriculoperitoneal shunt. The patient had completed chemotherapy with vincristine and carboplatin 2.5 years previously.

**FIGURE 4:**
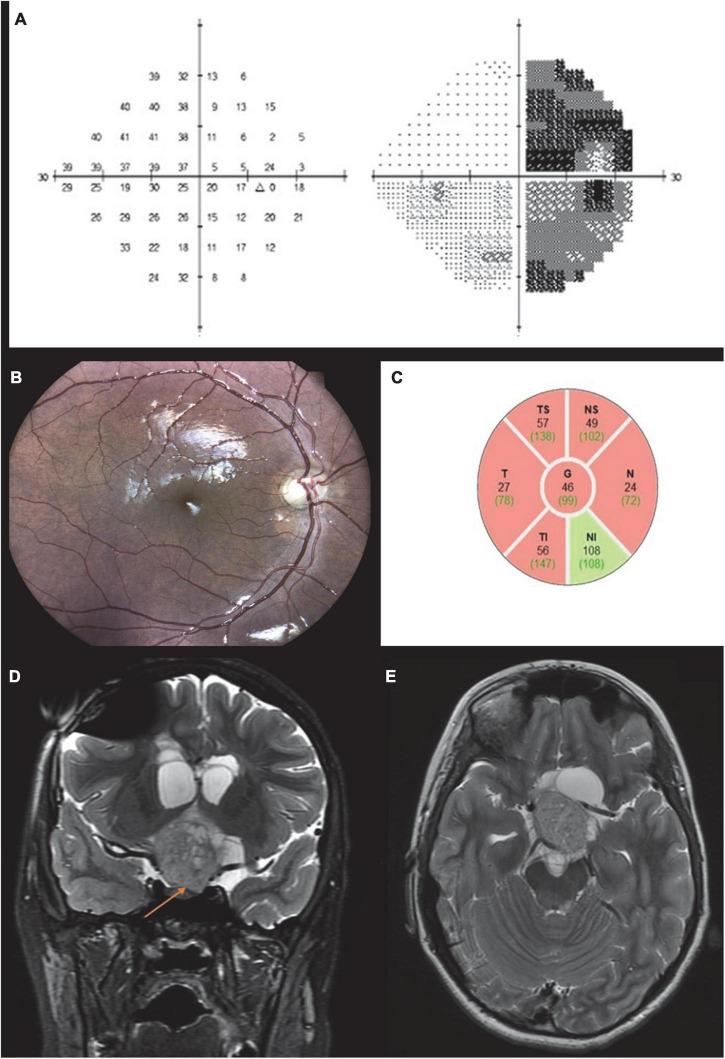
Case 4. Automated perimetry demonstrating temporal hemianopia in the right eye **(A)**. Brain MRI T2 coronal **(B)** and axial images **(C)** showing a large chiasmatic lesion extending to the left optic tract and left pre chiasmatic optic nerve causing compression of the third ventricle. Fundus color photograph shows bow tie atrophy of the right optic disc **(D)**. OCT scan of the right eye showing diffuse RNFL thinning **(E)**.

On examination, BCVA was 6/12 in the right eye and counting fingers in the left eye. Results of partial Ishihara color test were 6/10 in the right eye and none in the left eye. The left eye was RAPD + 3 positive, and there was bilateral limited elevation of gaze in both eyes. Confrontational visual field tests revealed a temporal field defect in the right eye, and automated perimetry demonstrated right hemianopia (Figure 4A). On slit-lamp examination, anterior segments and intraocular pressure were normal, and no Lisch nodules were seen. Fundus examination revealed bow-tie atrophy of the right optic disc (Figure 4B) and diffuse pallor of the left optic disc. A differential diagnosis of chiasmal defect (bitemporal) or optic tract defect (right hemianopsia) was suggested by the optic disc appearance. OCT showed diffuse thinning of the RNFL in the right eye, more prominent in the temporal and nasal quadrants, a pattern compatible with OTL (Figure 4C).

Brain MRI performed 1 month before presentation of the patient demonstrated a large space-occupying lesion affecting the left optic nerve and chiasm (Figures 4D,E), with no progression compared to previous MRI scans. A new scan did not demonstrate an optic tract lesion.

### Case 5

**FIGURE 5:**
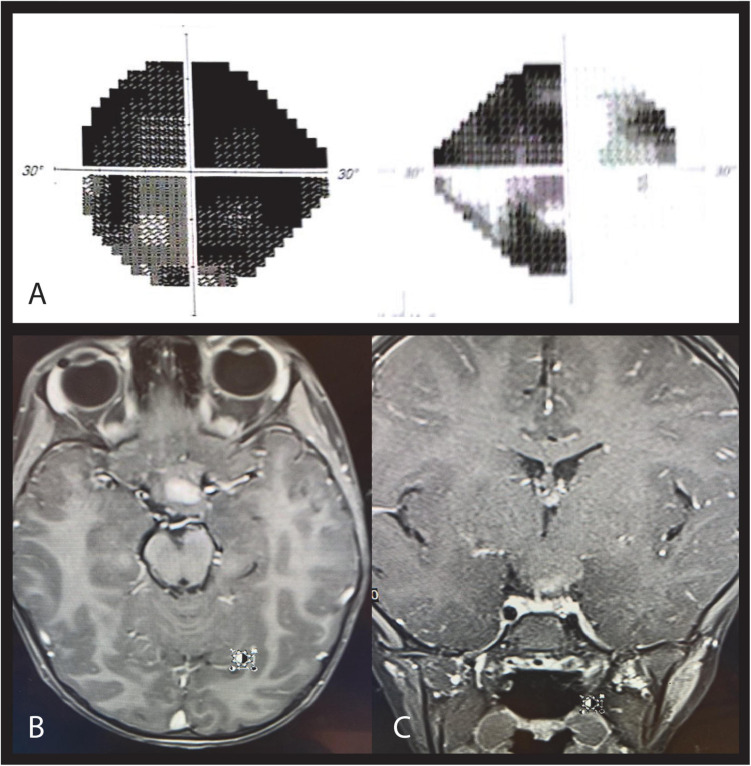
Case 5. Automated perimetry demonstrating a nasal defect respecting the vertical meridian in the right eye and a severely damaged field on the left **(A)**. MRI T1 post contrast axial **(B)** and coronal **(C)** scans showing a large chiasmatic hypothalamic enhancing lesion extending to the left optic tract and the left pre-chiasmatic optic nerve.

A 5-year-old girl with neurofibromatosis type 1 and a known chiasmal glioma involving the left optic nerve and left optic tract presented with decreased vision. She had already started vincristine and carboplatin chemotherapy. Visual acuity was 6/6 in the right eye and 6/120 in the left eye. RAPD + 3 due to severe optic neuropathy was noted in the left eye (overcoming left optic tract involvement). Results of Ishihara color test were normal in the right eye and null in the left eye. Confrontation visual field examination showed preference to the left field. Surprisingly, Humphrey visual field test, which she was undergoing for the first time, demonstrated a nasal defect respecting the vertical meridian in her right eye (Figure 5A). This subjective field was not in line with the other objective measurements of RGC loss and optic tract damage. The latter, together with RNFL measurements of 76 μm in the right eye and 46 μm in the left eye, suggested left chiasmal syndrome involving the left optic nerve, chiasm, and left optic tract. Comparison of the clinical findings with high quality fundus imaging clearly revealed that the optic atrophy represented the true pattern of left OTL with damage to the right field. MRI scans confirmed the diagnosis, showing a chiasmatic hypothalamic lesion extending to the left optic tract and the left pre-chiasmatic optic nerve (Figures 5B,C).

### Case 6

**FIGURE 6:**
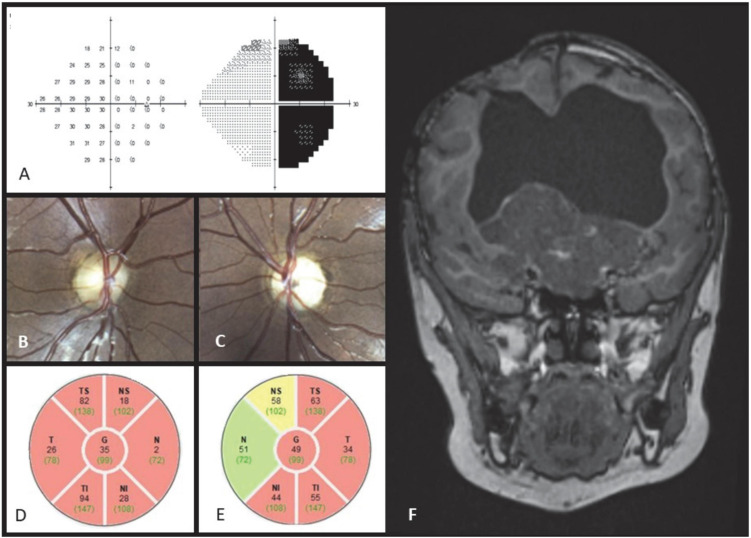
Case 6. Automated perimetry demonstrating a temporal hemianopia in the right eye **(A)**. Fundus color photograph shows bow tie atrophy of the right optic disc **(B)** and diffuse atrophy of the left optic disc **(C).** OCT scan showing bilateral diffuse RNFL thinning, which is more profound in the nasal and temporal quadrants of the right optic disc **(D,E)**. MRI T1 post contrast coronal scan showing massive suprasellar enhancing lesion and severe hydrocephalus **(F)**.

A 14-year-old boy with a known, longstanding left optic atrophy due to chiasmatic pilocytic astrocytoma attended our clinic. He previously underwent several neurosurgical procedures, a VP shunt and completed chemotherapy course 7 years earlier. On examination, visual acuity was 6/7.5 in the right eye and no light perception (NLP) in the left eye. Visual field confrontation test showed right visual field defect in his right eye, which was also demonstrated in Humphry perimetry test (Figure 6A). Albeit left optic tract damage, RAPD was positive in the left eye. This was due to severe left optic atrophy that was more dominant than the left optic tract damage. Ishihara test was normal in the right eye.

On fundoscopy the right optic disc revealed bow-tie atrophy (Figure 6B), while the left optic disc appeared diffusely atrophic (Figure 6C).

OCT RNFL of the right optic disc showed global RNFL thinning (averaging 35 μ), which was more profound in the nasal and temporal quadrants—compatible with relative bow tie atrophy pattern (Figure 6D). OCT RNFL of the left disc demonstrated thinning of the temporal and inferior quadrants (averaging 49 μ, Figure 6E). MRI showed a massive suprasellar space occupying lesion (SOL) and severe hydrocephalus (Figure 6F).

This case emphasizes the clinical importance of OCT and fundoscopy in identifying the bow tie atrophy pattern in the diagnosis of optic tract syndrome, particularly in cases in which one of the discs has previous severe damage.

## Discussion

OTLs are underdiagnosed (13, 14), and their underlying pathology can be life-threatening (tumor, vascular aneurysm). Prompt and accurate diagnosis is paramount. In the present study, we described 6 cases of OTL. We found that by systematically organizing the clinical, imaging, and visual field data, now available in almost every outpatient clinic, we were able to reliably rule out causes of homonymous hemianopia other than OTL and reach an accurate diagnosis. This method is particularly valuable in children, as visual fields are often unreliable and fundus exam for assessing optic disc pallor may be difficult. Although visual field testing in children can be challenging, we have found that following simple training in confrontational visual fields, some young children are able to perform automated perimetry reliably. Sita standard or Sita Fast 24-2 automated perimetry algorithm is used in our center, providing for a quick test with reliable and repeatable results. OCT scan is faster to acquire, which makes it a relatively reliable imaging modality in young children. In this study the reliability of the OCT scans was evaluated by signal strength values, as well as ruling out any imaging artifacts which may be present. The signal strength in all cases was 6/10 or higher. In adult patients, when diffuse axonal damage is suspected post trauma or tumor, the accurate involvement of the optic tract can be made without specific tractography on MRI. Although chiasmal lesions are unique in terms of the bitemporal field defect, when monocular visual loss occurs, it is almost impossible to differentiate between chiasmal and OTL based on visual fields alone. High quality optic disc color photography, now available in many ophthalmology emergency departments (15, 16), simplifies the diagnosis of OTL even in cases in which other imaging modalities are unfeasible, as in case 6 of the present study. Matching the disc appearance and RNFL thickness with optic nerve head OCT scans can demonstrate the pathognomonic bow-tie appearance of OTL.

OTL-induced optic tract syndrome may be the presenting sign of the intracranial pathology. In the past, the bow-tie-shaped atrophy could be identified only by experienced neuro-ophthalmologists and was commonly missed by others (1). The tremendous advances made in OCT technology combined with high quality optic disc fundus photographs acquired in seconds with advanced eye tracers, have greatly improved diagnostic accuracy. Furthermore, OCT derived ganglion cell maps may substitute the need of automated visual fields which are difficult to obtain in children. Lloyd-Smith et al. (11) reported that the clinical bow-tie sign is caused by the combination of papillomacular bundle and nasal RNFL loss. The optic tract contains uncrossed fibers originating from the temporal retina of the ipsilateral eye and crossed fibers from the nasal retina of the contralateral eye. The decussating fibers enter the optic nerve head at its nasal and temporal parts, and their atrophy results in the characteristic bow tie sign (3, 17, 18).

Our study shows that the straightforward complementary relations between RNFL and RGC loss on OCT with fundus photographs, visual field tests, and MRI scans in patients with OTL can assist less experienced ophthalmologists in clinical management. The close association between functional and anatomical parameters using OCT has been confirmed in numerous reports (19, 20). Ocular ultrasonography was not included in the study design and suggested algorithm, and may be considered in future studies. Although OTL rarely cause hydrocephalus or secondary papilledema, there is no doubt that US can contribute to the detection of swollen disc and differentiate pre chiasmatic lesions from retro-chiasmatic such as OTL (6, 7).

The ability to demonstrate RGC loss by OCT and to photograph the fundus, even in very young children, dramatically improved the ability to diagnose OTLs. The RGC loss demonstrated by OCT has a characteristic topographic pattern in line with visual field defects that respect the vertical meridian. These imaging modalities, together with high quality disc photographs, are helpful in reaching an accurate diagnosis (21).

OTL is a relatively rare clinical entity, and previous publications present case series with various etiologies as summarized in Table 1. Monteiro and Hokazone (22) described two cases in young patients, one due to trauma and the other a diagnosed tumor. Kanamori et al. (23) reported trauma, arterio-venous malformation, and two cases of malignancy resulting in right homonymous hemianopia due to left OTL. In our series, we report 6 patients of different ages, sex, and etiologies. Although head trauma is more common than tumors, optic tract injuries are not well documented in such cases.

**TABLE 1:** Clinical and OCT findings in patients with OTL: literature review.

					RNFL reduction on OCT	
Author, year	No. patients	Age (yr)/sex	Sex	OTL etiology	Ipsilateral eye	Contralateral eye
Tatsumi et al. (10)	1	20	F	Trauma	INF	TEMP, NAS
Monteiro and Hokazone (22)	2	23	M	1 Trauma	SUP, INF	TEMP, NAS
		29		1 Macroadenoma		
Hsu et al. (9)	8	37.7 ± 12.8	4 F	7 Trauma	SUP, INF, TEMP	TEMP, NAS, SUP, INF
		(mean)	4 M	1 PVL		
Kanamori et al. (23)	4	25.2 ± 4.4	2 F	1 Glioma	SUP, INF	TEMP, NAS
		(mean)	2 M	1 Metastasis		
				1 AVM		
				1 Trauma		
Gabilondo et al. (4)	1		F	MS	SUP, INF	NAS
Lloyd-Smith et al. (11)	1	49	M	NA	SUP, INF, TEMP	TEMP, NAS
Goto et al. (24)	8	40.4 ± 14.5 (mean)	1 F	Trauma		
			7 M			
Goto et al. (8)	1	25	M	Trauma	SUP, INF	TEMP, NAS
Present study, 2021	6	20.67 ± 18.47 (mean)	2 F	1 Trauma	SUP, INF	TEMP, NAS
			4 M	2 Astrocytoma		
				3 Glioma		
All studies	32	32.73 ± 15.41 (mean)	11 F	20 Trauma		
			21 M	8 SOL		
				1 AVM		
				1 PVL		
				1 MS		
				1 unavailable		

INF, inferior; NAS, nasal; SUP, superior; TEMP, temporal; AVM, arteriovenous malformation; MS, multiple sclerosis; PVL, periventricular leukomalacia.

In, Tatsumi et al. (10) reported on the role of OCT in a patient with OTL. In this study, we described a similar case of a 26-year-old woman with head trauma from a motor vehicle accident. Together, the findings emphasize the importance of diagnosing homonymous hemianopsia after trauma. Only one relevant, relatively large, case series (8 patients) has been published to date (9). An additional comparative study of 8 cases described the use of OCT to measure macular RNFL thickness in patients with OTL (24).

Study limitations include not using ocular ultrasonographical examination of the optic nerve which requires specifically trained medical personnel, and is not available in most clinics. We do not believe adding US to the algorithm may improve its use, but are confident that it can aid in differentiation of retro chiasmal lesions from optic neuropathy anterior to the chiasm in other cases.

## Conclusion

In conclusion, this case series examined 6 cases of long-standing OTL. The diagnosis of this rare condition is based on three classical clinical findings: positive RAPD, homonymous hemianopsia, and bow-tie-shaped atrophy of the optic discs. These may be revealed by careful examination, even when the OTL is not clearly visible on neuroimaging scans. This study showed that OCT can quantitatively detect RNFL loss and demonstrate the characteristic bow-tie optic disc pattern, thereby serving as a useful and reliable tool for the diagnosis of OTL. We recommend reviewing the clinical optic disc color photographs, visual fields, OCT scans, and MRI in sequence in cases of suspected OTL in order to simplify and improve the clinician’s diagnostic capabilities.

## Data availability statement

The original contributions presented in the study are included in the article/supplementary material, further inquiries can be directed to the corresponding author/s.

## Ethics statement

The studies involving human participants were reviewed and approved by the Bnai Zion Medical Center, Haifa, Israel. Written informed consent from the participants’ legal guardian/next of kin was not required to participate in this study in accordance with the national legislation and the institutional requirements.

## Author contributions

IM-P and NG-C were responsible for the conception and design. NC-S, IM-P, JL, and NG-C were responsible for acquisition of the data. NC-S, IM-P, NG-C, and AZ were responsible for the analysis and interpretation of the data, drafted the manuscript, and revised the manuscript for intellectual content. JL revised the neuroimaging. All authors approved the final version.
